# DPYD*6 plays an important role in fluoropyrimidine toxicity in addition to DPYD*2A and c.2846A>T: a comprehensive analysis in 1254 patients

**DOI:** 10.1038/s41397-019-0077-1

**Published:** 2019-02-06

**Authors:** Marzia Del Re, Saverio Cinieri, Angela Michelucci, Stefano Salvadori, Fotios Loupakis, Marta Schirripa, Chiara Cremolini, Stefania Crucitta, Cecilia Barbara, Angelo Di Leo, Tiziana Pia Latiano, Filippo Pietrantonio, Samantha Di Donato, Paolo Simi, Alessandro Passardi, Filippo De Braud, Giuseppe Altavilla, Claudio Zamagni, Roberto Bordonaro, Alfredo Butera, Evaristo Maiello, Carmine Pinto, Alfredo Falcone, Valentina Mazzotti, Riccardo Morganti, Romano Danesi

**Affiliations:** 1 Clinical Pharmacology and Pharmacogenetics Unit, Department of Clinical and Experimental Medicine, University of Pisa, Pisa, Italy; 2 Medical Oncology Division and Breast Unit, Civil Hospital, Brindisi, Italy; 3 Medical Genetics Unit, Department of Laboratory Medicine, Azienda Ospedaliero-Universitaria Pisana, Pisa, Italy; 4 Epidemiology and Health Services Research Department, Institute of Clinical Physiology, National Research Council (CNR), Pisa, Italy; 5 Medical Oncology Unit, Istituto Oncologico del Veneto IRCCS, Padova, Italy; 6 Medical Oncology Unit, Department of Translational Research and New Technologies in Medicine, University of Pisa, Pisa, Italy; 7 Medical Oncology Unit, Civil Hospital, Livorno, Italy; 8 Medical Oncology Unit, Civil Hospital, Prato, Italy; 9 Medical Oncology Unit, Casa Sollievo della Sofferenza IRCCS, San Giovanni Rotondo, Italy; 10 Medical Oncology Unit, Fondazione IRCCS Istituto Nazionale dei Tumori, Milano, Italy; 11 Medical Oncology Unit, Istituto Scientifico Romagnolo per lo Studio e la Cura dei Tumori IRCCS, Meldola, Italy; 12 Medical Oncology Unit, Department of Human Pathology, University of Messina, Messina, Italy; 13 Medical Oncology Unit, Addarii Institute of Oncology, S. Orsola-Malpighi Hospital, Bologna, Italy; 14 Medical Oncology Unit, Department of Oncology, ARNAS Garibaldi, Catania, Italy; 15 Medical Oncology Unit, Department of Oncology, Civil Hospital, Agrigento, Italy; 16 Medical Oncology Unit, Arcispedale Santa Maria Nuova IRCCS, Reggio Emilia, Italy; 17 Statistics Applied to Clinical Trials Unit, Azienda Ospedaliero-Universitaria Pisana, Pisa, Italy

**Keywords:** Cancer genomics, Predictive markers

## Abstract

Dihydropyrimidine dehydrogenase (*DPYD*) is a highly polymorphic gene and classic deficient variants (i.e., c.1236G>A/HapB3, c.1679T>G, c.1905+1G>A and c.2846A>T) are characterized by impaired enzyme activity and risk of severe adverse drug reactions (ADRs) in patients treated with fluoropyrimidines. The identification of poor metabolizers by pre-emptive *DPYD* screening may reduce the rate of ADRs but many patients with wild-type genotype for classic variants may still display ADRs. Therefore, the search for additional *DPYD* polymorphisms associated with ADRs may improve the safety of treatment with fluoropyrimidines. This study included 1254 patients treated with fluoropyrimidine-containing regimens and divided into *cohort 1*, which included 982 subjects suffering from gastrointestinal G≥2 and/or hematological G≥3 ADRs, and *cohort 2* (control group), which comprised 272 subjects not requiring dose reduction, delay or discontinuation of treatment. Both groups were screened for *DPYD* variants c.496A>G, c.1236G>A/HapB3, c.1601G>A (DPYD*4), c.1627A>G (DPYD*5), c.1679T>G (DPYD*13), c.1896T>C, c.1905 + 1G>A (DPYD*2A), c.2194G>A (DPYD*6), and c.2846A>T to assess their association with toxicity. Genetic analysis in the two cohorts were done by Real-Time PCR of DNA extracted from 3 ml of whole blood. *DPYD* c.496A>G, c.1601G>A, c.1627A>G, c.1896T>C, and c.2194G>A variants were found in both *cohort 1* and *2,* while c.1905+1G>A and c.2846A>T were present only in *cohort 1*. *DPYD* c.1679T>G and c.1236G>A/HapB3 were not found. Univariate analysis allowed the selection of c.1905+1G>A, c.2194G>A and c.2846A>T alleles as significantly associated with gastrointestinal and hematological ADRs (*p* < 0.05), while the c.496A>G variant showed a positive trend of association with neutropenia (*p* = 0.06). In conclusion, c.2194G>A is associated with clinically-relevant ADRs in addition to the already known c.1905+1G>A and c.2846A>T variants and should be evaluated pre-emptively to reduce the risk of fluoropyrimidine-associated ADRs.

## Introduction

Fluoropyrimidines are the most widely used chemotherapeutic agents for the treatment of many solid tumors, including gastrointestinal, head and neck, pancreas, and breast cancers [1]. Indeed, 5-fluorouracil (5-FU) and its prodrug capecitabine are the backbone of many combination chemotherapy regimens. Despite their clinical benefit, fluoropyrimidines are associated with adverse drug reactions (ADRs), including gastrointestinal and hematological toxicities and hand-foot syndrome (HFS), which may also be life-threatening [2]. ADRs may limit treatment effectiveness, because they impose modification of treatment schedules and/or their discontinuation. Therefore, there is a critical need for the identification of biomarkers predictive of drug-related toxicities, particularly in patients given adjuvant therapy [3]. Fluoropyrimidine metabolism involves numerous enzymes with many intermediate metabolites, but the rate-limiting step is dependent on dihydropyrimidine dehydrogenase (DPD), which metabolizes at least 80% of the administered dose of 5-FU or capecitabine into 5-fluoro-5,6-dihydrouracil (5-FDHU) [4]. If DPD is inactive or has reduced activity, the amount of 5-FU for anabolic activation increases, leading to 5-FU-related ADRs [4]. The major cause of DPD deficiency is the presence of mutations within the encoding gene *DPYD*, affecting splicing process, gene transcription and enzyme activity [5]. Many *DPYD* variants have been discovered [6–9], but most of them do not impair enzyme activity or their functional effect is unclear, with the exception of the splice site mutation in intron 14 (c.1905+1G>A, DPYD*2A) and the non-synonymous variant c.2846A>T (p.D949V), strongly associated with partial or complete loss of enzymatic activity and severe ADRs [10, 11]. Numerous efforts have been made to investigate the best approach to assess DPD deficiency and reduce the risk of toxicity [12] but, despite a strong laboratory rationale and cost-effectiveness of genotyping [13], the issue is still debated as contrasting recommendations on the implementation of *DPYD* analysis in clinical practice have been issued [14–16], fueling a debate on the usefulness of this test in the management of patients who are candidates to fluoropyrimidine treatment [17–19]. For these reasons, this study was designed to provide further evidence on the role of *DPYD* assessment by evaluating a large cohort of patients to discover which mutations should be tested to reduce the risk of ADRs and avoid unjustified costs of screening extremely rare *DPYD* genotypes.

## Materials and methods

### Study design and patients

Recruitment of patients started in October 2011 and ended in September 2017 and included a total of 1254 subjects. The study evaluated the possible association of the following *DPYD* variants selected on the basis of their occurrence in the general population and/or known to be involved in treatment-related ADRs: c.496A>G, c.1236G>A/HapB3, c.1601G>A (DPYD*4), c.1627A>G (DPYD*5), c.1679T>G (DPYD*13), c.1896T>C, c.1905+1G>A (DPYD*2A), c.2194G>A (DPYD*6), and c.2846A>T with ADRs requiring dose modifications, treatment delay or discontinuation. The population of 1254 subjects comprised a group of 982 patients (*cohort 1*) given fluoropyrimidine-based regimens to treat gastrointestinal, pancreatic, head and neck and breast cancers and suffering from G≥3 hematological or G≥2 gastrointestinal ADRs (CTCAE v.4). Overall, gastrointestinal toxicity is much less manageable than hematological ADRs, which are short lasting with fluoropyrimidines and do not usually require treatment with myeloid growth factors. On the contrary, starting from G2, gastrointestinal toxicity substantially impacts on the quality of life of patients and frequently requires dose modifications [20]. Patients received their first cycle of treatment at standard dosing and regimens as per current best practice guidelines; if irinotecan was also indicated, UGT1A1 analysis was performed and only subjects carrying the UGT1A1*1 or *1/*28 genotypes were included in *cohort 1*. Patients carrying the UGT1A1*28/*28 were excluded because of the high risk of developing gastrointestinal/hematological toxicities [21]. The same *DPYD* variants examined in *cohort 1* were also examined in a control population of 272 subjects (*cohort 2*) displaying optimal tolerability to treatment (no toxicity, dose reduction, treatment delay or discontinuation) to better define which *DPYD* variants are associated with clinically-relevant ADRs. Pharmacogenetic analysis was performed by real-time PCR by using the TaqMan® SNP Genotyping Assay (Life Technologies, Carlsbad, CA).

The study was approved by the Ethics Committee of Pisa University Hospital and conducted in accordance with the principles of the Declaration of Helsinki; all patients gave their signed informed consent before blood collection and DNA analysis.

### Statistical analysis

Categorical data were described by absolute and relative frequencies, whereas quantitative data were reported as mean and standard deviation. The association between *DYPD* variants and ADRs was evaluated by *χ*-^2^ test and odds ratio was also calculated. To compare the relative frequencies, *z*-test for two proportions was applied. Finally, all risk factors significantly influencing ADRs in the univariate analysis were assessed together in a binary logistic regression model as multivariate analysis. The results of the regression model were calculated by Wald test and expressed using odds ratio. A *p*-value < 0.05 was considered significant. All analyses, descriptive and inferential, were performed by the IBM SPSS statistics version 24.

## Results

A total of 1254 patients were enrolled in the study; 539 (43.0%) patients were male and 715 (57.0%) female, median age was 62 years (*cohort 1* interquartile range [IQR]: 14; *cohort 2* IQR: 10). A detailed description of patients is reported in Table 1. Since age and performance status have a significant impact on the occurrence of ADRs at the univariate analysis, the genotypic analysis was adjusted for these variables.

**Table 1:** Characteristics of patients of *cohorts 1 and 2*

Characteristics	Statistics	
	Cohort 1	Cohort 2
Patients	982	272
Gender (M/F)	392/590 (39.9/60.1)	147/125 (54/46)
Age (years)	63.9 ± 9.8	58.7 ± 7.4
Race	Caucasian	Caucasian
Disease		
Colorectal cancer	740 (75.4)	130 (47.8)
Gastric cancer	193 (19.6)	12 (4.4)
Breast cancer	49 (5.0)	130 (47.8)
Treatment^a^		
FU-LV (De Gramont regimen)	170 (17.3)	0 (0)
Capecitabine	210 (21.4)	92 (33.8)
FOLFIRI	182 (18.5)	0 (0)
FOLFOX-4	190 (19.3)	130 (47.8)
FOLFOXIRI	54 (5.5)	0 (0)
CAPOX	160 (16.3)	0 (0)
TPF	0 (0)	0 (0)
XELIRI	8 (0.7)	0 (0)
EOX^b^	8 (0.8)	50 (18.4)
ADRs		
Gastrointestinal	Grade≥2	
Nausea/Vomiting	16%	0 (0)
Diarrhea	39.7%	0 (0)
Stomatitis	14%	0 (0)
Dermatological	Grade≥2	
Hand-foot syndrome	9.3%	0 (0)
Hematological	Grade≥3	
Fever	2.2%	0 (0)
Leucopenia	12.3%	0 (0)
Neutropenia	17.4%	0 (0)
Febrile neutropenia	4.7%	0 (0)
Anemia	4.2%	0 (0)
Thrombocytopenia	5.8%	0 (0)

^a^Abbreviations listed as per NCI Thesaurus v. 16.08e (release 2016-08-29)

^b^Epirubicin, oxaliplatin, capecitabine

*Cohort 1* (Table 1) consisted of 982 patients (590 females [60.1%] and 392 males [39.9%]); gastrointestinal (G≥2) and hematological toxicities (G≥3) were present in 69.7% and 46.6% of patients, respectively. A control group of 272 patients (147 males [54%] and 125 females [46%], *cohort 2*) receiving standard doses of fluoropyrimidine-based therapies, without dose reduction, delay or discontinuation, were also enrolled (Table 1). The frequencies of c.496A>G, c.1601G>A, c.1627A>G, c.1896T>C, c.1905+1G>A, c.2194G>A and c.2846A>T alleles are reported in Table 2. The c.1679T>G and c.1236G>A/HapB3 variants were not found neither in *cohort 1* nor in *cohort* *2*. The comparison between the two cohorts demonstrated that IVS14+1GA/AA, c.2194GA/AA, c.2846AT/TT were significantly higher in *cohort 1* than in *cohort 2*: 6.2% vs. 0% (*p* < 0.0001), 19.7% vs. 11.8% (*p* = 0.004) and 2.4% vs. 0% (*p* = 0.020), respectively (Table 2). The statistical analysis showed that IVS14+1GA and AA genotypes were significantly associated with diarrhea (*p* = 0.001), alopecia (*p* = 0.007), febrile neutropenia (*p* < 0.0001), and thrombocytopenia (*p* = 0.012). c.2194GA/GG were associated with stomatitis (*p* = 0.053), leucopenia (*p* = 0.003), and thrombocytopenia (*p* = 0.049). Finally, c.2846AT/TT were associated with diarrhea (*p* = 0.02, Table 3). The strong association of c.1905+1G>A and c.2846A>T with ADRs was also demonstrated by the absence of c.1905+1A and c.2846T variant alleles in *cohort 2* (Table 2). Borderline associations of c.496AG/GG (*p* = 0.068) and c.1905+1GA/AA (*p* = 0.061) with neutropenia and of 2194GA/AA (*p* = 0.062) with febrile neutropenia were found. On the contrary, c.1601G>A, c.1627A>G and c.1896T>C played no role in fluoropyrimidine toxicities (Table 3). At univariate analysis, the incidence of ADRs was lower in patients treated with fluoropyrimidines alone or in association with oxaliplatin *vs*. all other treatments (Table 1S). However, at multivariate analyses, *DPYD* variants were confirmed as independent factors of ADRs irrespective of treatments received (Table 4).

**Table 2:** Type and frequencies of *DPYD* genotypes in *cohorts 1* and *2*

	Heterozygous + homozygous mutants (%)		
SNPs	*Cohort 1*	*Cohort 2*	*p*-value
c.496A>G	23.8	18	0.052
c.1601G>A	9.3	6.2	0.136
c.1627A>G	32.6	39.7	0.035^a^
c.1679T>G	Not found	Not found	Not found
c.1896T>C	3.5	4.8	0.415
IVS14+1G>A	6.2	0	**<0.0001**
c.2194G>A	19.7	11.8	**0.004**
c.2846A>T	2.4	0	**0.020**
c.1236G>A/HapB3	Not found	Not found	Not found

^a^Higher frequency in *cohort 2*

**Table 3:** *DPYD* variants and associations with ADRs

ADRs		c.496A>G	c.1601G>A	c.1627A>G	c.1679T>G	c.1896T>C	c.1905+1G>A	c.2194G>A	c.2846A>T	c.1236G>A/HapB3
Nausea/vomiting	*p*-value	0.773	0.287	0.598	Not found	0.836	0.418	0.830	0.074	Not found
Diarrhea	*p*-value	0.613	0.074	0.347	Not found	0.111	**0.001** (OR 2.317)	0.725	**0.020** (OR 2.602)	Not found
Stomatitis	*p*-value	0.205	0.079	0.236	Not found	0.396	0.076	0.053 (OR 1.514)	0.674	Not found
Dermatitis	*p*-value	0.215	0.618	0.482	Not found	0.969	0.152	0.152	0.749	Not found
Alopecia	*p*-value	0.406	0.848	0.689	Not found	0.655	**0.007** (OR 4.239)	0.886	0.486	Not found
Leucopenia	*p*-value	0.100	0.464	0.979	Not found	0.294	0.265	**0.003** (OR 1.895)	0.467	Not found
Neutropenia	*p*-value	0.068 (OR 1.408)	0.638	0.396	Not found	0.339	0.061 (OR 1.757)	0.127	0.658	Not found
Febrile neutropenia	*p*-value	0.470	0.366	0.745	Not found	0.245	**<0.0001** (OR 4.135)	0.062 (OR 1.838)	0.272	Not found
Anemia	*p*-value	0.620	0.729	0.653	Not found	0.561	0.081	0.595	0.960	Not found
Thrombocytopenia	*p*-value	0.612	0.080	0.647	Not found	0.984	**0.012** (OR 2.686)	**0.049** (OR 1.796)	0.592	Not found
HFS	*p*-value	0.102	0.269	0.911	Not found	0.659	0.081	0.371	0.940	Not found
Fever	*p*-value	0.513	0.909	0.816	Not found	0.392	0.478	0.084	0.475	Not found

**Table 4:** Multivariate analysis of toxicity risk factors. *p* and OR values are indicated. OR > 1 if associated with a *p*-value < 0.05 indicates a toxicity risk factor

ADRs		c.496A>G	IVS14+1G>A	c.2194G>A	c.2846A>T	Treatment combinations other than fluoropyrimidines ± oxaliplatin^a^
Diarrhea	*p*-value		**0.001** (OR 2.408)		**0.017** (OR 2.777)	**0.008** (OR 0.656)
Stomatitis	*p*-value		0.067 (OR 1.836)	**0.049** (OR 1.536)		**<0.0001** (OR 0.442)
Leukopenia	*p*-value			**0.003** (OR 1.958)		**<0.0001** (OR 0.339)
Alopecia	*p*-value		**0.012** (OR 4.370)			**0.024** (OR 0.344)
Neutropenia	*p*-value	**0.054** (OR 1.452)	**0.042** (OR 1.894)			**<0.0001** (OR 0.301)
Febrile neutropenia	*p*-value		**<0.0001** (OR 4.241)	0.060 (OR 1.879)		0.079 (OR 0.555)
Thrombocytopenia	*p*-value		**0.011** (OR 2.863)	**0.040** (OR 1.875)		**<0.0001** (OR 0.319)

^a^FOLFIRI, FOLFOXIRI, TPF, XELIRI, EOX

## Discussion

An extensive search of genetic variants of *DPYD* associated with enzyme deficiency and poor-metabolizer status has been performed and several genotypes were identified [5, 8, 22]. In agreement with three meta-analyses [10, 23, 24], our study confirmed the well-known role of c.1905+1G>A and c.2846A>T in fluoropyrimidine-associated ADRs. An additional meta-analysis also found an association between severe ADRs and the non-synonymous variant c.1679T>G (DPYD*13) as well as with the synonymous variant c.1236G>A in complete linkage with HapB3 [25], a haplotype containing three intronic polymorphisms (IVS5+18G>A, IVS6+139G>A and IVS9-51T>G) [26, 27]. Several other variants have been associated with fluoropyrimidine toxicities, including c.257C>T, c.1850C>T [28], c.2509-2510insC, c.1801G>C, c.680G>A [29], c.85T>C (p.R29C) [30], and c.496A>G (p.M166V) [31]; however, due to the lack of confirmatory studies, their association with toxicity remains unproven.

The present study found a significant association between the non-synonymous variant c.2194G>A (p.V732I, DPYD*6) with ADRs by fluoropyrimidines. The results of the present work provide additional information on the debate on this pharmacogenetic marker. Despite the c.2194A allele seems to be relatively common, conflicting results have been reported concerning its influence on DPD activity and association with clinically-relevant ADRs. Some studies did not assign a role to c.2194G>A in the occurrence of fluoropyrimidine toxicity [32, 33] and in silico analysis demonstrated a normal enzyme activity [8]. A study found that c.2194G>A showed weak evidence for association with reduced DPD activity in African-American patients; c.2194GA patients displayed a 29% reduction in DPD activity compared to the wild-type, although the linkage with c.557A>G (p.Y186C) may have played a prominent role [34]. In the study by Schwab et al. [35] the role of c.2194G>A was not considered significant, but this result may have been affected by the small number of carriers of this variant. Another study on the role of selected *DPYD* variants on treatment tolerability showed no association between G≥3 toxicity and c.2194G>A, but, also in this case, this result may have been affected by the small group of patients [36].

On the contrary, the secondary analysis of the Pan-European Trials in Alimentary Tract Cancer (PETACC-8) study provided evidence of the association of c.2194G>A variant with clinically-relevant ADRs in FOLFOX4-treated patients [37] and the same result was observed in the TOSCA randomized trial that enrolled colon cancer patients given 3 or 6 months of either FOLFOX-4 or XELOX adjuvant chemotherapy [38]. In particular, the work by Boige et al. examined a cohort of 1545 patients and found a significant association of ADRs with c.2194G>A variant [37]. The statistical analysis revealed a correlation between G≥3 ADRs by 5-FU and c.2194G>A (OR = 1.7; *p* < 0.001); in more detail, G≥3 hematologic adverse events (OR = 1.9) and G≥3 neutropenia (OR = 1.8) were associated with c.2194G>A [37]. Data generated within a clinical study have the clear advantage of being obtained in a selected, homogeneous population with strict follow-up. Our study has the limitation of having enrolled a heterogenous population but what can be viewed as a limitation turns to be an important confirmation of PETACC-8 results in different clinical settings, thus demonstrating the usefulness of c.2194G>A screening. It should be noted that the data of the present study are in accordance not only with the results provided by the PETACC-8 trial but also with the previously published biomarker analysis of TOSCA trial, which found a significant association between c.2194G>A and time to neutropenia [38]. A smaller study found a relationship of c.2194G>A with leukopenia (OR = 8.17) and neutropenia (OR = 2.78) [39] and an additional work also positively associated this variant with diarrhea [27]. Finally, a meta-analysis conducted on seven cohort studies, with a total of 946 colorectal cancer patients receiving 5-FU chemotherapy, found a significant association between the c.2194G>A polymorphism, bone marrow suppression (*p* < 0.001) and gastrointestinal ADRs (*p* < 0.05) [23]. Although other mutations of *DPYD* may represent a risk for patients [14, 37, 40] their extremely low frequency does not suggest their inclusion in routine preemptive screening.

Recommendations are available on which variants to test and which dose adjustement of fluoropyrimidines should be adopted and include c.1236G>A/HapB3, c.1679T>G, c.1905+1G>A, and c.2846A>T [41, 42]. The updated guideline on DPYD genotyping [42] is an extremely valuable instrument to apply targeted genotyping in current clinical laboratory practice. It is not surprising that it includes only mutations (i.e., IVS14+1G>A, c.2846A>T, HapB3, and c.1679T>G) supported by a substantial amount of clinical data and established association with toxicities, while it does not recommend novel variants like c.2194G>A which still lack of clear clinical information and/or controversial data are provided due to small groups of patients examined in some studies. Despite the large number of published works, the present study is one of the few addressing the issue of *DPYD* variants and treatment safety in a large population. We purposely included patients given various protocols containing fluoropyrimidines to assess the impact of *DPYD* polymorphisms in different settings and validate the role of gene variants independently of the drugs combined with fluoropyrimidines. A direct comparison between the same regimens would have been statistically more correct and this is a weakness of the present study. However, due to the multitude of drug combinations containing fluoropyrimidines and disease settings, a much larger patient population should have been enrolled. Nonetheless, the role of major variants (IVS14+1G>A, c.2846A>T) has been confirmed in this study, despite the heterogeneity of clinical settings, and the importance of c.2194G>A is further documented by the present work.

It is still a matter of debate when to screen subjects candidate to fluoropyrimidine treatment and if the therapeutic drug monitoring (TDM) has a role in optimizing drug doses. In selected patients in whom dose adaptation is difficult, measurement of 5-FU and of its major metabolite 5-FDHU can be performed. TDM is an extremely useful approach but turnaround time is longer than SNP genotyping and pre-analytical issues may limit its widespread use. In some centers, patients are prospectively screened and dose reductions are made, if necessary. Unfortunately, *DPYD* screening is still not universally accepted, although it has been demonstrated to be cost-effective [43], and several subjects are examined only after an ADR has occurred, thus abolishing the advantage of a preemptive genotyping to reduce the deleterious consequences of administering a fluoropyrimidine in a poor metabolizer.

In conclusion, the present article provides evidence that c.2194G>A should be examined in addition to well-known deleterious variants; a dose reduction of 20% in homozygous variant patients and a close monitoring of heterozygous subjects for ADRs are thus advisable.

## Supplementary information

Table 1S
