# Supplementary material for: DPYD*6 plays an important role in fluoropyrimidine toxicity in addition to DPYD*2A and c.2846A>T: a comprehensive analysis in 1254 patients
Source: Pharmacogenomics J. 2019 Feb 6;19(6):556–63. doi: 10.1038/s41397-019-0077-1 (PMC6867961; doi:10.1038/s41397-019-0077-1)
Supplement: Supplementary file 1 — Table 1S [file 41397_2019_77_MOESM1_ESM.docx]

**Table 1S.** ADRs associated with fluoropyrimidines ± oxaliplatin *vs.* all other combinations^a^

|  | **ADRs (%)** | |  |
| --- | --- | --- | --- |
| **ADRs** | **Fluoropyrimidines ± oxaliplatin** | **All other combinations** | ***p*-value** |
| **Nausea/vomiting** | 15 | 19.6 | 0.136 |
| **Diarrhea** | 37.5 | 47.5 | 0.012 |
| **Stomatitis** | 11.4 | 22.5 | **<0.0001** |
| **Dermatitis** | 3.1 | 2.9 | 0.936 |
| **Alopecia** | 1.4 | 3.9 | 0.042 |
| **Leucopenia** | 9.1 | 22.5 | **<0.0001** |
| **Neutropenia** | 13.2 | 33.3 | **<0.0001** |
| **Febrile neutropenia** | 4.1 | 6.9 | 0.134 |
| **Anemia** | 2.8 | 8.3 | **<0.0001** |
| **Thrombocytopenia** | 4.2 | 11.8 | **<0.0001** |
| **Hand-foot syndrome** | 4.9 | 2.9 | 0.299 |
| **Fever** | 0.7 | 4.9 | 0.003 |

^a^FOLFIRI, FOLFOXIRI, TPF, XELIRI, EOX
